# Multiple Approaches at Admission Based on Lung Ultrasound and Biomarkers Improves Risk Identification in COVID-19 Patients

**DOI:** 10.3390/jcm10235478

**Published:** 2021-11-23

**Authors:** Rubio-Gracia Jorge, Sánchez-Marteles Marta, Garcés-Horna Vanesa, Martínez-Lostao Luis, Ruiz-Laiglesia Fernando, Crespo-Aznarez Silvia, Peña-Fresneda Natacha, Gracia-Tello Borja, Cebollada Alberto, Carrera-Lasfuentes Patricia, Pérez-Calvo Juan Ignacio, Giménez-López Ignacio

**Affiliations:** 1 Internal Medicine Department, Clinical Hospital “Lozano Blesa”, 50009 Zaragoza, Spain; marta.sanchez15@yahoo.es (S.-M.M.); vanesa_garces@hotmail.com (G.-H.V.); fruizl@unizar.es (R.-L.F.); silviacrespoaz@gmail.com (C.-A.S.); bcgracia@salud.aragon.es (G.-T.B.); jiperez@unizar.es (P.-C.J.I.); 2 Aragon Health Research Institute, 50009 Zaragoza, Spain; lmartinezlos@salud.aragon.es (M.-L.L.); npfrosyoly@gmail.com (P.-F.N.); albertocebollada@hotmail.com (C.A.); pcarreralasfuentes@gmail.com (C.-L.P.); igimenez@unizar.es (G.-L.I.); 3 Immunology Department, Clinical Hospital “Lozano Blesa”, 50009 Zaragoza, Spain; 4 Center for Biomedical Research of Aragon, 50009 Zaragoza, Spain; 5 Biocomputation Unit, Center for Biomedical Research of Aragon, 50009 Zaragoza, Spain; 6 Biomedical Research Networking Center in Hepatic and Digestive Diseases (CIBERehd), 28005 Madrid, Spain; 7 School Medicine, Zaragoza University, 50009 Zaragoza, Spain

**Keywords:** lung ultrasound, COVID-19, ST-2

## Abstract

Background: Risk stratification of COVID-19 patients is fundamental to improving prognosis and selecting the right treatment. We hypothesized that a combination of lung ultrasound (LUZ-score), biomarkers (sST2), and clinical models (PANDEMYC score) could be useful to improve risk stratification. Methods: This was a prospective cohort study designed to analyze the prognostic value of lung ultrasound, sST2, and PANDEMYC score in COVID-19 patients. The primary endpoint was in-hospital death and/or admission to the intensive care unit. The total length of hospital stay, increase of oxygen flow, or escalated medical treatment during the first 72 h were secondary endpoints. Results: a total of 144 patients were included; the mean age was 57.5 ± 12.78 years. The median PANDEMYC score was 243 (52), the median LUZ-score was 21 (10), and the median sST2 was 53.1 ng/mL (30.9). Soluble ST2 showed the best predictive capacity for the primary endpoint (AUC = 0.764 (0.658–0.871); *p* = 0.001), towards the PANDEMYC score (AUC = 0.762 (0.655–0.870); *p* = 0.001) and LUZ-score (AUC = 0.749 (0.596–0.901); *p* = 0.002). Taken together, these three tools significantly improved the risk capacity (AUC = 0.840 (0.727–0.953); *p* ≤ 0.001). Conclusions: The PANDEMYC score, lung ultrasound, and sST2 concentrations upon admission for COVID-19 are independent predictors of intra-hospital death and/or the need for admission to the ICU for mechanical ventilation. The combination of these predictive tools improves the predictive power compared to each one separately. The use of decision trees, based on multivariate models, could be useful in clinical practice.

## 1. Introduction

Severe acute respiratory syndrome coronavirus type 2 (SARS-CoV-2) causes COVID-19 disease [1,2]. This infectious disease is capable of causing multi-organic involvement [3,4], bilateral viral pneumonia, and sometimes, early respiratory distress syndrome in adults (ARDS), with the need for mechanical ventilation in about 8% of total COVID-19 hospitalizations [2,5].

COVID-19 pathophysiology has several well-differentiated phases [2]. During the first week, a viral picture occurs that later gives way to a pro-inflammatory state [3], influenced by cytokine storm and thrombotic phenomena [6]. This situation can last for months in some cases, which is called post-COVID-19 syndrome [7,8]. Because of this unusual evolution for an infectious disease [2], sometimes, clinicians find it difficult to identify patients at higher risk of admission or in need of early intensive care [1]. Therefore, the availability of clinical tools to stratify risk and design specific diagnostic–therapeutic strategies is essential to improve outcomes.

Risk-stratification tools for COVID-19 were initially based on the analysis of baseline clinical characteristics through retrospective cohort studies during the first pandemic wave [1,9]. As we gathered greater knowledge about the disease and clinical experience accumulated [5,6,8], research and practice moved from clinical data to more complex analysis based on biomarkers [10,11,12,13], imaging [14,15], or functional study of the main affected organs [16].

We have previously shown the predictive value of lung ultrasound (LUS) [17] and biochemical biomarkers [18]. Now we aim at developing a multidimensional approach for risk stratification in COVID-19 patients. Our hypothesis is that the addition of lung ultrasound and laboratory biomarkers to a proven clinical score (PANDEMYC score) [9] increases its predictive ability to detect worse outcomes.

## 2. Materials and Methods

### 2.1. Study Design

This is a unicenter, prospective study carried out between July and October 2020 in the Infectious Diseases and Internal Medicine service of a tertiary university hospital. Inclusion and exclusion criteria have been previously published [17]. In summary, they were patients admitted with confirmed COVID-19 infection, respiratory symptoms, stable from the respiratory point of view (without requiring initial admission to the ICU or mechanical ventilation), and without advanced cognitive impairment. Exclusion criteria were (1) previous intensive care unit (ICU) admission; (2) refusal of the patient to participate; (3) functional dependence (Barthel index < 50 points); (4) moderate/severe cognitive impairment (Pfeiffer scale); (5) advanced COPD (forced expiratory volume in 1 s < 30%) or a history of emphysema and/or pulmonary fibrosis; or (6) active cáncer.

During the first 72 h of admission, vital signs were recorded. Estimated PAFI (oxygen saturation/FiO_2_ supplied) and Borg scale were used as indirect markers of respiratory function. The assessment was complemented with routine blood tests (CBC, biochemistry, coagulation, and gas tests). Additional blood samples were collected with patient consent and stored at −80 °C in the Biobank of the Aragon Health Research Institute (IIS Aragon) until analysis.

### 2.2. Risk Prediction through Basic Clinical and Analytical Parameters

PANDEMYC score [9] was selected as the reference predictive model in this study. It is a prediction model for COVID-19 patients, based on basic clinical and laboratory data at admission, that has been demonstrated to predict in-hospital death of COVID-19 patients [9]. This model was chosen based on the following arguments: first, its creation was based on a cohort of patients from our same country and with similar characteristics; second, score is calculated from nine variables that are easy to obtain in routine clinical practice, even if some of them are missing; and third, the tool shows excellent power in predicting a hard target such as in-hospital death (AUC = 0.88) [9].

### 2.3. Point-of-Care Lung Ultrasound and Biomarkers

LUS was performed to identify and quantify lung damage caused by SARS-CoV-2 infection. LUS was developed following a previously described protocol [17]. We developed a lung injury score (LUZ-score) based on the recognition of four simple patterns (scoring from 0 to 4) in twelve different thoracic areas with a final score between 0 and 48 points [17].

Soluble ST-2 (sST2) was selected as an indirect biomarker for lung damage. Soluble ST2 determinations were carried out from serum aliquots previously inactivated for the SARS-CoV-2 virus with 1% Triton-X100. Serum sST2 was quantified by enzyme-linked immunosorbent assay (ELISA), following the instructions from the manufacturer (DY523B, R&D Systems Europe Ltd., Minneapolis, MN, USA).

### 2.4. Primary and Secondary Outcomes

In-hospital death from any cause and/or the need for admission to the ICU for the administration of mechanical ventilation was selected as the primary endpoint. Secondary endpoints were: (1) need to increase O_2_ therapy during the first 72 h. (2) Need to increase medical treatment (increase initial corticosteroids dose, add remdesivir or other biological therapies) during the first 72 h. (3) Length of hospital stay in those patients who did not reach primary endpoint. (4) Combined endpoint including need to increase O_2_ or COVID-19 therapy during the first 72 h after admission.

### 2.5. Statistical Analysis

An initial descriptive analysis of all clinical variables was carried out. Continuous variables are reported as mean with standard deviation (SD) or median with interquartile range (IQR), whereas qualitative variables are expressed as frequencies and percentages. The relationship between qualitative variables was evaluated with Chi-square (χ^2^) test. Student *t*-test or Mann–Whitney U test was employed for comparing means of two independent groups, and ANOVA or Kruskal–Wallis test was used when the qualitative variable had more than two categories. Normality was tested using Kolmogorov–Smirnov test.

Logistic regression models were constructed, and odds ratios (ORs) and 95% confidence intervals (CIs) were calculated. The discriminatory accuracy of models was evaluated using the area under the receiver operating characteristics (ROC) curve (AUC) or c-index [19]. Then, ROC curves were compared two by two [20]

Classification trees (CART) were constructed for predicting primary outcomes based on PANDEMYC score [9], point-of-care lung ultrasound (LUZ score) [17], and baseline sST2 concentrations. The rpart algorithm [21] was used for generating decisions trees. Pruning and tuning parameters were applied to optimize the predictive model by avoiding an over-complex tree and thus increase the model’s accuracy. Ten-fold was used to estimate out-of-sample accuracy, given the constraint on data availability and avoiding over-fitting issue. To reduce variability, multiple rounds of cross-validation were performed using different partitions, and validation results were combined over rounds to estimate model’s performance [22].

For all tests, a two-sided *p* < 0.05 was considered statistically significant. Statistical analysis was carried out with Statistical Package for the Social Sciences (SPSS, version 24.0 for Windows. IBM Corp., Armonk, NY, USA). CART was constructed with caret library from R package (Version 6.0-88) and rpart2 algorithm (Max Kuhn (2021)).

The study was approved by the regional research ethics committee (CEICA, Ref. PI20/248, 13 May 2020) and met the basic requirements of the ethics guidelines of Helsinki Declaration.

## 3. Results

### 3.1. Baseline Characteristics

From the 151 patients initially recruited, 144 patients were finally included (seven blood samples were not available for ELISA). The mean age was 57.5 ± 12.78 years, and 60.4% were males. Comorbidities with higher prevalence were hypertension (37.5%), smoking (33.6%), dyslipidemia or previous statins treatment (29.2%), and diabetes (17.4%). (Table 1).

### 3.2. Characteristics according to the PANDEMYC Score at Admission

The PANDEMYC score median was 243 points (52). Patients with the highest PANDEMYC score (over 75 percentile, >266 points) were older, with a higher prevalence of comorbidities such as hypertension, heart failure, or chronic kidney disease (CKD). Creatinin-phosphokinase (CPK), lactate deshidrogenase (LDH), C-reactive protein (CRP), or Interleuquin-6 (IL-6) concentrations were significantly higher among those patients over 75 percentile. Soluble ST2 concentrations were also higher in this group, although significance was not reached. Patients with a PANDEMYC score over the percentile of 75 also showed a higher rate of lung injury by LUS or estimated PAFI (Table 1).

### 3.3. Outcomes and Multivariable Logistic Regression Model

The primary endpoint (in-hospital death and/or admission to ICU after the first 72 h of admission) was reached in 15 patients (10.4%). One patient died in the ICU, and fourteen were transferred to ICU for mechanical ventilation. The median length of stay in those patients who did not reach the primary endpoint was 7 days (5). An increase in oxygen supply during the first 72 h after admission was administered to 34.1% of patients, and almost 40% needed an update in medical COVID-19 therapy (either increasing intravenous dexamethasone dose or adding new medical treatments). (Supplementary Table S1).

The primary endpoint was significantly higher among those patients with a PANDEMYC score > 75 percentile (22.2% vs. 6.5%; *p* = 0.012), as was the need to increase O_2_ therapy during the first 72 h (53.8% vs. 25.0%; *p* = 0.005). The length of stay was also longer among this group (11 days vs. 7 days; *p* ≤ 0.001) (Supplementary Table S1).

**Figure 1:**
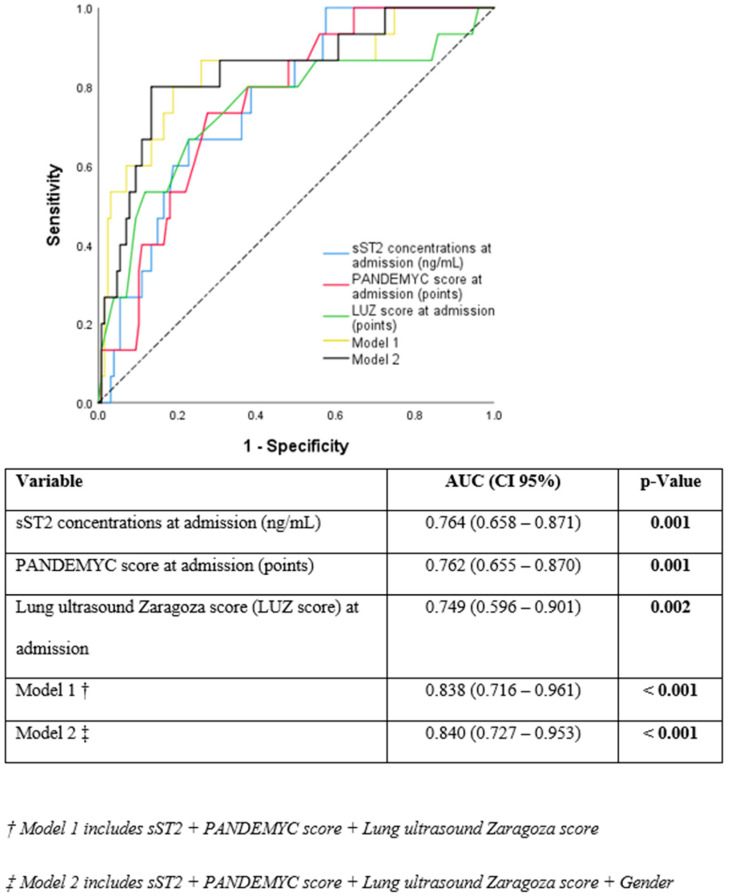
Receiver operating characteristic curves of the different models analyzed.

The PANDEMYC score and LUS and sST2 concentrations at admission were identified as independent predictors for the primary endpoint in univariable logistic regression analysis (Table 2). When comparing predictive capacity, sST2 showed very similar values (AUC =0.764 (0.658–0.871); *p* = 0.001), as PANDEMYC score (AUC = 0.762 (0.655–0.870); *p* = 0.001) and LUS (LUZ-score) (AUC = 0.749 (0.596–0.901); *p* = 0.002) (Figure 1). However, the combination of these three diagnostic tools (PANDEMYC, lung ultrasound and sST2) in a multivariable logistic regression model significantly improved the predictive risk capacity (AUC = 0.838 (0.716–0.961); *p* ≤ 0.001). The addition of gender to the model did not cause any further gain (AUC = 0.840 (0.727–0.953); *p* ≤ 0.001) (Figure 1 and Supplementary Table S2).

### 3.4. Decision Diagrams Based on Classification Trees

We propose two different predictive risk models for the primary endpoint, from statistical simulation and taking into account the previously mentioned prediction tools (PANDEMYC score, point of care lung ultrasound, and baseline sST2 concentrations).

**Figure 2:**
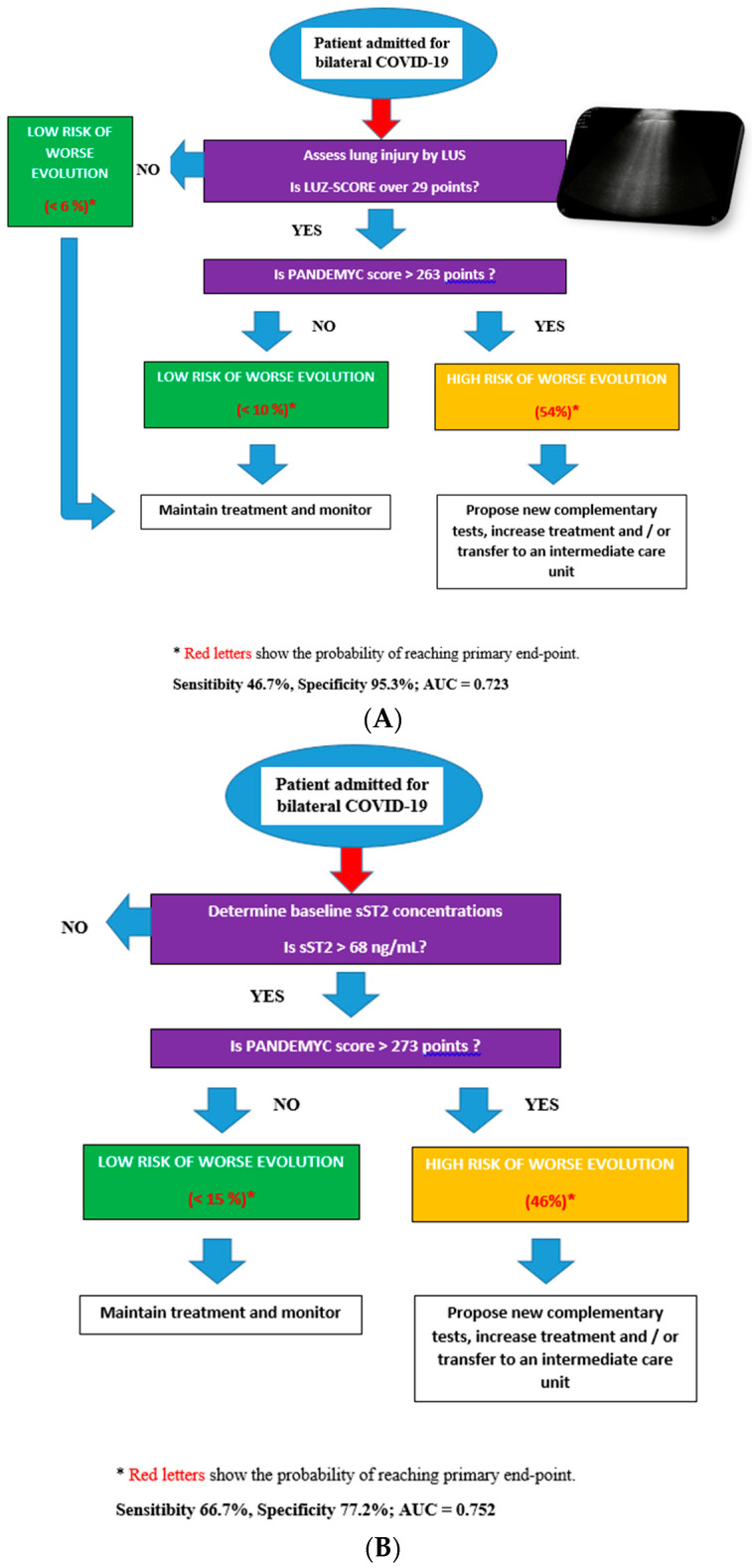
(**A**): Decisions diagrams proposed based on LUZ-score and PANDEMYC score. (**B**): Decisions diagrams proposed based on sST2 and PANDEMYC score.

The first model includes an initial evaluation by LUS. If LUZ-score is equal to or below 29 points, the probability of achieving the primary endpoint is 6%. However, if LUS involvement is significant at admission (above 29 points), the model suggests a second evaluation with a PANDEMYC score to assess risk. In this case, if this value is higher than 263 points, the probability of developing the primary endpoint is 54%, with a sensitivity of 46.7% and a specificity of 95% (AUC = 0.723) (Figure 2A).

The second model suggests an initial assessment of sST2 concentrations. If sST2 is lower than 68 ng/mL, the probability of reaching the primary endpoint is 5%. However, if baseline sST2 is higher than 68 ng/mL, the model suggests a second evaluation with a PANDEMYC score (cut-off 273 points). If sST2 is higher than 68 ng/mL, and the PANDEMYC score is >273 points, the probability of reaching the primary endpoint is 54% (Sensibility 66.7%, Specificity 77.2%; AUC = 0.752) (Figure 2B).

## 4. Discussion

In this study, we validated the PANDEMYC score for the primary endpoint of in-hospital death and/or ICU admission during the first 72 h. Furthermore, we confirmed our initial hypothesis, showing that a multimodal assessment, based on biomarkers (sST2), LUS (LUZ-score), and PANDEMYC score, improves the risk-predictive capacity of each marker alone. The creation of a decision tree that combines clinical data, LUS, and biomarkers (sST2) in COVID-19 patients is unprecedented.

Identifying patients who may present serious complications after being admitted for COVID-19 is vital to improve the patient’s prognosis. Since COVID-19 started, several scoring scales as CURB-65, ISARIC [23], or pneumonia score index (PSI) have been validated for in-hospital death outcomes [24]. However, given that COVID-19 is a novel disease, having specific tools to improve clinical care is essential.

In our analysis, the PANDEMYC score [9] is able to identify a population with a higher prevalence of comorbidities and pro-inflammatory status inferred through a significant increase in concentrations of CPK, LDH, CRP, or lymphopenia (Table 1). In addition, IL-6 and sST2 concentrations were also higher in the group of patients with a higher PANDEMYC score, data that would confirm the implication of interleukins in the prognosis of patients. However, our results show that the predictive capacity of the PANDEMYC score is lower than previously described, probably because we selected a combined primary endpoint instead of a harder endpoint as in-hospital death alone, but also because our sample size was limited. That is why our study aims to improve the predictive capacity of the PANDEMYC score, providing objective tools such as lung ultrasound or novel biomarkers in this field.

LUS has been demonstrated to be useful to detect patients with worse outcomes [14,15,17,25,26] with similar accuracy as computed tomography [27]. According to our results, the predictive power of LUS (LUZ-score) [17] for the primary endpoint is similar to the PANDEMYC score (AUC 0.749 vs. AUC 0.762; *p* = 0.869). However, LUS has some disadvantages, as results can differ between observers or a high sensitivity but low specificity [17], a situation that can be explained given that pattern recognition (b lines), is common to other diseases, such as heart failure [28]. This argument makes a point of LUS as an ideal screening test, but with a high rate of false positives when trying to predict hard outcomes. Therefore, our proposal to combine prediction tools to increase statistical power seems reasonable.

Specifically, in this study, we propose to complement the PANDEMYC score and LUS with a novel biomarker that has been linked to lung injury in COVID-19 during admission (sST2). Soluble ST2 is the interleukin 33 (IL-33) receptor, and its concentrations have been associated with inflammatory phenomena and acute lung damage in processes of non-cardiogenic origin [29]. Since lung tissue is the most important target for SARS-CoV-2 [30], sST2 has been tested as a novel predictive biomarker in COVID-19 [18,31]. In our analysis, sST2 was the strongest predictor for the primary endpoint, on top of the PANDEMYC score and point-of-care lung ultrasound. (Figure 1, Table 2, and Supplementary Table S2). Now, since it is a biomarker also related to other processes—mainly heart failure [32,33] and myocardial involvement [34]—sST2 concentrations must be interpreted with caution in patients with COVID-19 because myocardial affection produced by COVID-19 [35] could increase basal levels of sST2. Furthermore, sST2 concentrations might change according to diurnal variation in some patients, and that situation should also be considered [36].

In summary, the combination of these three tests (PANDEMYC score, point-of-care lung ultrasound, and sST2 concentrations) is superior in improving the ability to predict risk at admission in patients with COVID-19 (Figure 1, Table 2, and Supplementary Table S2). These results are novel and could be transferred to routine clinical practice once validated or even lay the basis for clinical trials to standardize treatment based on objective data. Furthermore, our models consider as a primary objective not only mortality but also the need to enter the ICU for mechanical ventilation. This is an added value, especially if we take into account the AUCs reached (Table 2 and Supplementary Table S2). Most of the predictive models in which clinical variables [9] or lung ultrasound [37,38] are used only contemplate mortality as the primary outcome, when identifying those patients who require early intubation is probably more practical to improve care, and this fact makes our models more attractive.

In an attempt to make an approach to clinical practice, we propose two decision algorithms based on the creation of statistical multivariable models (Figure 2A,B). Both models propose an initial screening either with LUS or with sST2 and subsequently a second evaluation with the PANDEMYC score to identify patients at higher risk with similar results (Figure 2A,B). The simulation did not find an additional improvement of the prediction by combining the three variables to generate a decision tree, so both models (considering two of the three predictive tools analyzed) can be used indistinguishably. Selecting one or another model should be based on several factors. A patient with acute heart failure or a history of interstitial lung disease is likely to benefit more from a biomarker-based model (sST2), as a lung ultrasound could be biased. However, predictive models based on soluble biomarkers are more expensive, and they require having such a laboratory technique, which is not always available.

### Limitations

The results have been obtained from a single center and therefore cannot be extrapolated. Although clinical characteristics in our cohort were concordant with published data from other studies around the world and in our country [17,18], external validation is necessary to determine a prediction model’s reproducibility and generalizability to new and different patients. The sample size of the study was calculated based on the number of blood samples needed to analyze the sST2, and therefore the power of the multivariate model could have been underestimated. Lung ultrasound is a person-dependent technique, and therefore this fact could affect the final result. Finally, the decision trees have been elaborated from statistical models and would need to be validated in a different cohort.

## 5. Conclusions

PANDEMYC score, lung ultrasound, and sST2 concentrations upon admission for COVID-19 are independent predictors of intra-hospital death and/or the need for admission to the ICU for mechanical ventilation. The combination of these predictive tools improves the predictive power compared to each one separately. The use of decision trees, based on multivariate models, could be useful in clinical practice.

## Figures and Tables

**Table 1:** Baseline characteristics according to PANDEMYC score (tertiles) at admission.

Variable	TOTAL	*p* < 25 (<214 Points)	*p* 25 to 75 (214–266 Points)	*p* > 75 (>266 Points)	*p*-Value
Total size (N)	144				
Age (years) *	57.5 ± 12.8	42.7 ± 9.2	59.6 ± 9.3	68.1 ± 7.9	**<0.001**
Gender-Male (n (%))	87 (60.4)	21 (58.3)	42 (58.3)	24 (66.7)	0.471
Duration of symptom (days)	6.5 ± 3.3	6.6 ± 3.3	6.6 ± 3.3	6.0 ± 3.3	0.677
Time until COVID-19 confirmation (Days)	3 (7)	2 (6)	3 (7)	3 (8)	0.832
Comorbidities (n (%)):					
• Hypertension	54 (37.5)	4 (11.1)	28 (38.9)	22 (61.1)	**<0.001**
• Heart failure	4 (2.8)	0 (0.0)	1 (1.4)	3 (8.3)	**0.033**
• Dyslipidemia	42 (29.2)	7 (19.4)	15 (20.8)	20 (55.6)	**0.001**
• Coronary artery disease	5 (3.5)	1 (2.8)	3 (4.2)	1 (2.8)	1.000
• Diabetes	25 (17.4)	2 (5.6)	17 (23.6)	6 (16.7)	0.215
• History of smoking *	48 (33.6)	6 (16.7)	26 (36.1)	16 (45.7)	**0.010**
• COPD/Asthma	16 (11.1)				
• Atrial/flutter fibrillation	5 (3.6)	0 (0.0)	2 (2.9)	3 (8.3)	0.059
• CKD	7 (4.9)	1 (2.8)	1 (1.4)	5 (13.9)	**0.029**
Clinical variables					
• BMI (Kgs/m^2^)	28.9 (6.4)	30.2 (7.8)	29.1 (6.6)	28.2 (4.9)	0.568
• SBP (mmHg)	126.9 ± 16.7	124.6 ± 15.3	126.2 ± 17.9	130.5 ± 15.1	0.301
• DBP (mmHg)	77.2 ± 10.9	76.9 ± 11.4	76.5 ± 10.8	79.2 ± 10.5	0.480
• HR (bpm)	80.9 ± 12.8	83.1 ± 13.7	80.0 ± 13.4	80.5 ± 10.4	0.490
• Estimated PAFI (mmHg)	367 (92)	429 (74)	403 (94)	340 (76)	**0.001**
• Borg scale for dyspnea (points)	4 (6)	5 (6)	5 (4)	4 (5)	0.844
Laboratory:					
• Urea (mg/dL)	33 (19)	28 (16)	31 (14)	40 (23)	**0.002**
• Creatinine (mg/dL) *	0.94 (0.29)	0.82 (0.26)	0.89 (0.28)	1.05 (0.51)	**<0.001**
**Variable (Continue)**	**TOTAL**	***p* < 25**	***p* 25 to 75**	***p* > 75**	***p*-Value**
Laboratory:					
• Aspartate transaminase (U/L)	37 (27)	38 (48)	34 (20)	41 (21)	0.338
• Alanine transaminase (U/L)	31 (28)	40 (56)	31 (20)	28 (25)	0.175
• Creatin phophokinase (U/L)	94 (92)	103 (116)	83 (63)	129 (92)	**0.048**
• Lactate deshidrogenase (U/L)	306 (145)	282 (94)	306 (114)	369 (202)	**0.007**
• C-Reactive Protein (mg/L) *	63 (81)	38 (77)	53 (70)	91 (98)	**0.002**
• Ferritin (ng/mL)	707 (908)	682 (917)	710 (914)	699 (1022)	0.666
• Hemoglobin (g/dL) *	14.2 ± 1.5	14.3 ± 1.1	14.2 ± 1.6	14.1 ± 1.7	0.707
• Total leucocytes (×1000)	5.6 (3.1)	5.0 (1.9)	5.8 (3.6)	6.1 (3.1)	0.407
• Total lymphocytes (×1000) *	0.9 (0.7)	1.1 (0.6)	1.0 (0.6)	0.7 (0.5)	**0.019**
• Total platelets (×1000) *	173 (100)	189 (75)	176 (118)	147 (87)	**0.016**
• D-Dimer (ng/mL)	688 (633)	664 (560)	654 (519)	802 (820)	0.195
• Fibrinogen (mg/dL)	775 (208)	783 (193)	763 (212)	779 (243)	0.976
• Interleukine-6 (pg/mL)	40 (30)	39 (27)	29 (31)	50 (57)	**0.041**
• sST2 (ng/L)	53.1 (30.9)	49.3 (24.9)	50.8 (32.0)	62.1 (36.6)	0.060
X-rays (n (%))					0.192
• Normal	25 (17.9)	8 (22.9)	12 (16.9)	5 (14.7)	
• Unilateral consolidation	35 (25.0)	9 (25.7)	20 (28.2)	6 (17.6)	
• Bilateral consolidations	80 (57.1)	18 (51.4)	39 (54.9)	23 (67.6)	
Lung ultrasound (LUZ-score)	21 (10)	18 (12)	21 (10)	22 (10)	**0.024**
Therapies (n (%))					
• Colchicine	10 (6.9)	4 (11.1)	4 (5.6)	2 (5.6)	0.525
• Remdesivir	46 (31.9)	10 (27.8)	18 (25.0)	18 (50.0)	**0.026**
• Systemic corticosteroids	113 (78.5)	28 (77.8)	52 (72.2)	33 (91.7)	0.153
• Medium dose of corticosteroids (Dexametasone (mg))	6 (3)	6 (0)	6 (3)	6 (3)	0.156

* Variables included in PANDEMYC score.

**Table 2:** Univariable and multivariable logistic regression analysis for the primary endpoint (in-hospital death and/or need for ICU admission for mechanical ventilation).

Univariable			Multivariable		
Variable	OR (CI 95%)	*p*-Value	Variable	OR (CI 95%)	*p*-Value
PANDEMYC score (points)	1.03 (1.01–1.05)	**0.002**	PANDEMYC score (points)	1.02 (1.01–1.04)	**0.034**
sST2 (ng/mL)	1.02 (1.01–1.03)	**0.016**	sST2 (ng/mL)	1.02 (1.01–1.03)	**0.038**
LUZ-score (points)	1.13 (1.04–1.22)	**0.004**	LUZ-score (points)	1.12 (1.02–1.22)	**0.014**

## Supplementary Materials

The following are available online at https://www.mdpi.com/article/10.3390/jcm10235478/s1, Table S1: Outcomes by PANDEMYC score at baseline, Table S2: Receiver operating characteristic curves comparison.
