# Supplementary material for: Multiple Approaches at Admission Based on Lung Ultrasound and Biomarkers Improves Risk Identification in COVID-19 Patients
Source: J Clin Med. 2021 Nov 23;10(23):5478. doi: 10.3390/jcm10235478 (PMC8658110; doi:10.3390/jcm10235478)
Supplement: Supplementary file 1 [file jcm-10-05478-s001.zip › jcm-1450077-supplementary.pdf]

Journal of
Clinical Medicine

MDPI

Supplementary Materials

Table S1. Outcomes by PANDEMYC score at baseline (comparison between patients with PANDEMYC score > P75 and the rest of population).

Variable	TOTAL	≤P75	>P75	*p*-Value
**Primary outcome (n (%)):**				
• **ICU admission and/or death**	15 (10.4)	7 (6.5)	8 (22.2)	**0.012**
**Secondary outcomes:**				
• **Length of stay (days)**	7 (5)	7 (4)	11 (6)	**<0.001**
• **Necessity of higher O2 therapy at 48/72 h (n (%))**	47 (34.1)	25 (25.0)	14 (53.8)	**0.005**
• **Necessity to update COVID-19 treatment at 48/72 h (n (%))**	53 (37.9)	34 (34.0)	11 (39.3)	0.605
• **Necessity of higher O2 therapy or update COVID-19 treatment at 48/72 h (n (%))**	66 (48.5)	40 (40,4)	16 (61.5)	0.054

ICU: Intensive care unit. Bold results are statistically significant.

Table S2. Receiver operating characteristic curves comparison.

ROC Curves Compared	AUC Difference CI (95%)	*p*-Value
ST2 concentrations vs. PANDEMYC score	0.002 (-0.147–0.151)	0.983
ST2 concentrations vs. LUS (LUZ-score)	0,015 (-0.185–0.216)	0.880
ST2 concentrations vs. Model 1 †	-0.074 (-0.227–0.078)	0.339
ST2 concentrations vs. Model 2 ‡	-0.076 (-0.226–0.074)	0.321
PANDEMYC score vs. LUS (LUZ-score)	0.014 (-0.151–0.179)	0.869
PANDEMYC score vs. Model 1 †	-0.076 (-0.185–0.033)	0.171
PANDEMYC score vs. Model 2 ‡	-0.077 (-0.179–0.024)	0.134
LUS (LUZ-score) vs. Model 1 †	-0.090 (-0.168–-0.011)	**0.025**
LUS (LUZ-score) vs. Model 2 ‡	-0.091 (-0.180–-0.003)	**0.043**
Model 1 † vs.Model 2 ‡	-0.002 (-0.033–0.030)	0.923

† Model 1 includes sST2 + PANDEMYC score + lung ultrasound Zaragoza score. ‡ Model 2 includes sST2 + PANDEMYC score + lung ultrasound Zaragoza score + age. Bold numbers are statistically significant. LUS: lung ultrasound.
